# Comparative Analysis of MicroRNAs between Sporophyte and Gametophyte of *Porphyra yezoensis*

**DOI:** 10.1155/2012/912843

**Published:** 2012-09-26

**Authors:** Linwen He, Aiyou Huang, Songdong Shen, Jianfeng Niu, Guangce Wang

**Affiliations:** ^1^Key Laboratory of Experimental Marine Biology, Institute of Oceanology, Chinese Academy of Sciences (IOCAS), Nanhai Road 7, Qingdao 266071, China; ^2^School of Earth Science, Graduate University of Chinese Academy of Sciences, Yuquan Road 19, Beijing 100049, China; ^3^College of Life Sciences, Soochow University, Renai Road 199, Suzhou 215123, China

## Abstract

*Porphyra yezoensis* Ueda is an intertidal marine red algae that has received increasing attention as a model organism owing to its important role in biological research and the agronomic industry. The two generations of *Porphyra yezoensis*, the sporophyte and the gametophyte, have the same genome but show great differences in many aspects, including structural features, habitat, and gene expression. To identify miRNAs and their probable roles in *P. yezoensis* development, we constructed and sequenced libraries of small RNA from *P. yezoensis* sporophytes and gametophytes. The sequencing data were analyzed, and 14 miRNAs were identified, with only one common to these two samples. Our results show that *P. yezoensis* has a complex small RNA processing system containing novel miRNAs that have no identifiable homolog in other organisms. These miRNAs might have important regulatory roles in development of the different generations of *P. yezoensis*.

## 1. Introduction

*Porphyra yezoensis* Ueda is an intertidal marine red algae that has received increasing attention as a model organism owing to its important role in biological research and the agronomic industry [1]. *P. yezoensis* is one of the most valuable marine crops in the world and is cultivated widely in Asia, especially in Japan, China, and Republic of Korea [2]. As a red algae, whose relationship to other groups is by no means certain, *P. yezoensis* has many characteristics of lower eukaryotes, including the location of the small subunit of Rubisco in the chloroplast genome [3], the same accessory pigments as cyanobacteria [4], and the high degree of similarity between many of its genes and their homologs in bacteria. Owing to these features, red algae are believed to be original or degraded eukaryotic organisms [5].

*P. yezoensis* has a unique dimorphic life-cycle consisting of two generations, a microscopic diploid filamentous sporophyte and a macroscopic haploid foliate gametophyte, with completely different morphology [6]. The sporophyte and the gametophyte have the same genome but show great differences in many aspects, including structural features, habitat, and gene expression. The sporophyte is densely tufted with uniseriate filaments, whereas the gametophyte is monolayered. The sporophyte enters and germinates in shells, whereas the gametophyte lives on static substrates of the intertidal zone, experiencing stress caused by strong light, high temperature, and desiccation during low tide [7–9]. Analysis of expressed sequence tag (EST) groups generated from sporophytes and gametophytes of *P. yezoensis* found that only 22.5% of groups commonly occurred in both generations, indicating great differences in gene expression resulting in morphological differences between the two generations [10]. These characteristics prompted us to hypothesize that the gene expression regulators (e.g., microRNAs (miRNAs)) of the two generations might show different specificity.

miRNAs are important noncoding small RNAs (sRNAs) that can influence the output of quantity genes in eukaryotes by targeting mRNAs for translational repression or cleavage [11–13]. miRNAs have been widely studied only recently, but they are attracting a great deal of attention and are being studied in many organisms. miRNAs are believed to exist in animals, plants, and viruses with a high degree of conservation in each kingdom [14, 15]. The expression of miRNAs has a spatiotemporal pattern [11, 12, 16–18], and each of them influences the transcription and translation of specific genes [15]. miRNAs are of simple structure and have important roles in gene regulation in various processes [15], including developmental patterning, cell proliferation, tumor generation [19], stress resistance [19], auxin response [20, 21], fat metabolism, and miRNA biogenesis [22, 23]. miRNAs have been studied extensively in higher plants and in animals, and Liang et al. [24] identified miRNAs from the *P. yezoensis* sporophyte but the likely roles of miRNA in the development of different generations of *P. yezoensis *remain unknown.

In the present study, we constructed sRNA libraries from the sporophyte and the gametophyte of* P. yezoensis* then used high-throughput Solexa technology to deeply sequence the sRNAs. The sequencing data were analyzed, and miRNAs were identified from both samples studied. This study has provided insights into the expression and function of small silencing RNAs in *P. yezoensis*.

## 2. Materials and Methods

### 2.1. Culture of Sporophytes and Gametophytes of *P. yezoensis*

The *P. yezoensis* sporophytes and gametophytes of *P. yezoensis* were available in our laboratory. Both were cultured by constant aeration in Provasoli enriched seawater (PES) culture medium made with steam-sterilized local seawater supplemented with inorganic nutrients and vitamins (filter sterilized), with renewal of the culture medium every week. As the sporophytes and gametophytes grow well under different conditions, they were cultured at different temperatures and under different light intensities. The sporophytes were grown at 15°C under an illumination intensity of 50 *μ*mol photons m^−2^ s^−1^ with a 12 h dark/12 h light photoperiod. The gametophytes were induced from conchocelis; basically, a shell with *P. yezoensis* conchocelis was cultured at 26°C with PES medium in a 500 mL glass beaker under an illumination intensity of 20 *μ*mol photons m^−2^ s^−1^ with a 12 h dark/12 h light photoperiod. After several weeks, the shell was placed at 22°C and some fibers were placed on it for attachment of the conchospores. The fibers with conchospores were cultured at 10°C under an illumination intensity of 50 *μ*mol photos m^−2^ s^−1^ with a 12 h dark/12 h light photoperiod. Blades were selected randomly for further culture in 1-liter glass beakers. 

### 2.2. RNA Extraction, Library Construction, and Sequencing

**Figure 1:**
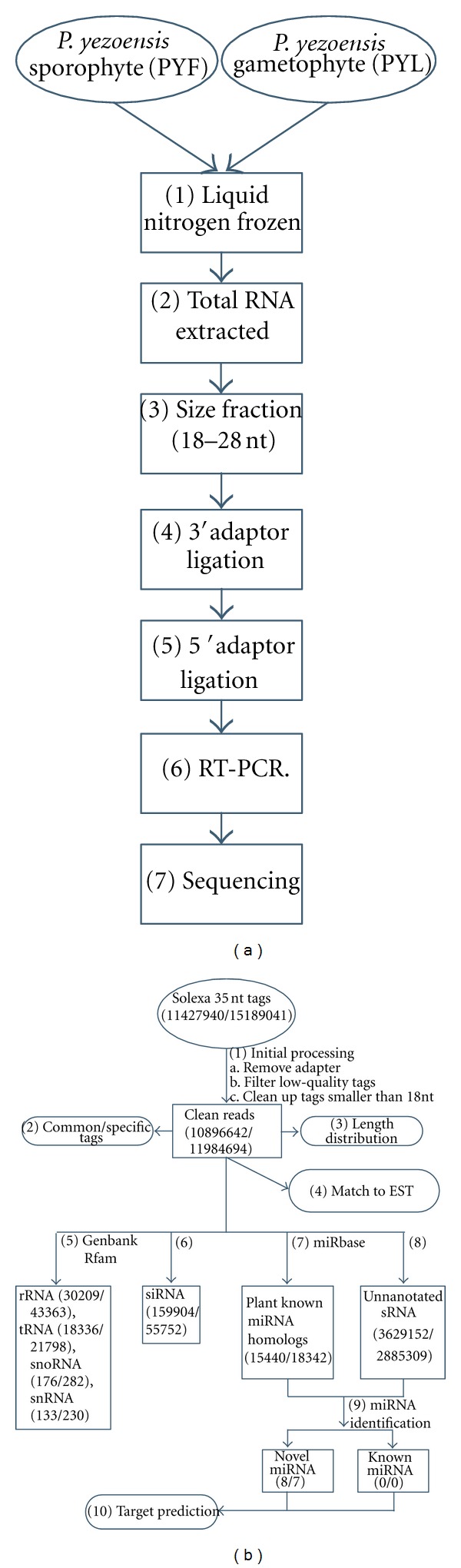
A flow chart of the procedure for sample preparation and sequencing and for the processing of reads. (a) A flow chart of the procedure for sample preparation and sequencing. (1) Sporophytes and gametophytes of* P. yezoensis *were frozen rapidly in liquid nitrogen and stored at −80°C before RNA extraction. (2) Total RNA was extracted using the Trizol reagent method. (3) RNA 18–28 nt fragments were gel-purified. (4) A 3′ adaptor was ligated to the 3′ end of sRNAs. (5) A 5′ adaptor was ligated to the 5′ end of sRNAs. (6) sRNAs were amplified by RT PCR. (7) Sequencing. (b) A flow chart of the procedure for processing reads; the numbers in parentheses represent the total reads from PYF and PYL, respectively. (1) Initial processing: remove adapter, filter out low-quality tags, and clean up tags smaller than 18nt. (2) Common/specific tags identified between samples. (3) Length distribution analysis of clean reads. (4) Clean reads matched to *P. yezoensis* EST sequences using SOAP [25]. (5) Clean reads compared to noncoding RNAs from GenBank and Rfam. (6) siRNA identified. (7) Plant miRNA homologs identified. (8) Annotated sRNAs. (9) miRNA identified by hairpin structure filtering. (10) Target prediction.

Total RNA was extracted with Trizol reagent according to the manufacturer's protocol (Invitrogen, USA). sRNAs with a size range of 18–28 nt were collected and sequenced. Basically, sRNAs were separated by electrophoresis in denaturing polyacrylamide gels, and fragments of 18–28 nt were recovered by gel purification. A pair of adaptors was ligated sequentially to their 5′- and 3′-ends, and the ligated sRNAs were used as template for cDNA synthesis, using the SuperScript II Reverse Transcription Kit (Invitrogen, USA). The cDNAs were then amplified by PCR, and the products were sequenced directly with a Solexa 1G Genome Analyzer (Figure 1).

### 2.3. Initial Processing of Reads

A perl script was used to complete the initial processing of reads. Low-quality tags were removed, 3′ adaptor sequences were trimmed, adaptor contamination tags were removed, and tags smaller than 18 nt were filtered out. The Short Oligonucleotide Analysis Package (SOAP) [25] was used to map the remaining sRNA sequences (clean reads) to the *P. yezoensis *EST sequences; all hits were reported and mismatch was not allowed. All the clean reads were aligned against noncoding RNA from Rfam (http://www.sanger.ac.uk/resources/databases/rfam.html) and GenBank (http://www.ncbi.nlm.nih.gov/) to identify noncoding RNAs (rRNA, tRNA, snRNA, and snoRNA) fragments, using blastn [26] with an *e*-value of 0.01 as cutoff. All the clean reads were compared to all known plant miRNAs available from miRBase (miRBase Sequence Database version 15; http://www.mirbase.org/) to identify homologs of known miRNAs (≤2 mismatches with other known miRNAs). Tags from clean reads were aligned with each other to identify potential small interfering (siRNA) candidates; the two perfectly complementary sRNAs with 2 nt overhangs at the 3′-end were considered to be siRNA. All clean reads were classified according to their identity with the sRNA categories mentioned above. In the case that an sRNA was mapped to more than one category, the following priority rule was adopted: rRNA and so forth (in which GenBank > Rfam) > known miRNA > siRNA [27].

### 2.4. miRNA Identification

miRNAs were identified by structure filtering. As the genome of *P. yezoensis* was not sequenced, EST sequences were scanned to identify potential hairpin regions for homologs of known miRNAs and the remaining nonannotated sRNAs (Figure 1). sRNAs with more than one read and ≤20 perfect matches to EST sequences were folded with 300 nt of upstream and downstream flanking sequences and examined for secondary structures to identify potential miRNAs on the basis of the criteria described in the following. Precursors with minimum free energy (MFE) ≤−18 kcal mol^−1^ checking by Mfold [28, 29], ≥16 bp and ≤4 bulges or asymmetries between miRNA and miRNA*, with space between miRNA and miRNA* ≤300 nt, mature sequence length 18–25 nt, and a flank sequence length of 20 nt, were considered to be potential* P. yezoensis* pre-miRNAs.

### 2.5. miRNA Target Prediction

As the genome of *P. yezoensis* has not been sequenced, EST sequences were used to detect potential miRNA targets with parameters and conditions according to the criteria suggested earlier [30, 31]. Basically, ≤4 mismatches between the sRNA and the target; ≤2.5 mismatches in positions 1–12; no mismatch in positions 10 or 11; no adjacent mismatch in positions 2–12 (counting from the 5′-end of the miRNAs and G-U bases as 0.5 mismatch). Additionally, an MFE of ≥74% of the MFE of the miRNA bound to its perfect complement was required for the miRNA/target duplex. 

### 2.6. Experimental Verification of the Expression of *P. yezoensis* miRNAs

RT PCR was used to detect the expression of *P. yezoensis* miRNAs. cDNAs were synthesized using the NCode VILO miRNA cDNA Synthesis Kit (Invitrogen) according to the manufacturer's protocol. The cDNA was used for PCR amplification of *P. yezoensis* miRNAs. The sense primers were designed according to each miRNA and the antisense primer was the universal primer supplied in the cDNA synthesis kit. 

## 3. Results

### 3.1. Library Construction, Sequencing, and Initial Processing of Reads

**Figure 2:**
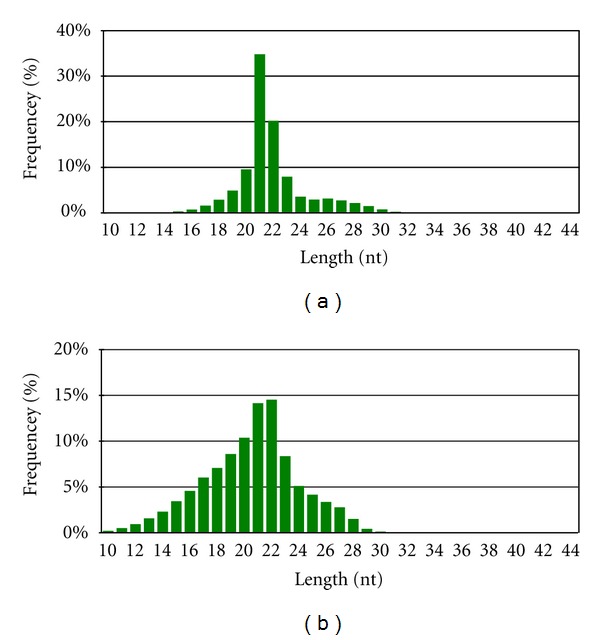
Length distributions of unique sRNA sequences in *P. yezoensis.* The length occurrence of each unique sequence read was counted: (a) PYF; (b) PYL.

In order to identify miRNAs and their likely roles in *P. yezoensis *development, we constructed and sequenced sRNA libraries from the *P. yezoensis *sporophyte (PYF) and gametophyte (PYL). The two runs yielded a set of 26,616,981 total signatures. After filtering out low-quality data and elimination of adaptor contamination (Figure 1), we obtained sRNAs with a distribution of lengths ranging from 10 to 43 nt, with sequences of 21-22 nt as the major component (Figure 2). miRNAs were commonly larger than 17 nt, so we removed sequences smaller than 18 nt and obtained 10,896,642 and 11,984,694 total sequences, representing 3,853,350 and 3,025,076 unique, although sometimes partially overlapping, clean reads from PYF and PYL, respectively (Table 1). Of these unique sequences, ~75% (2,879,648) and ~74% (2,235,970) were sequenced only once, indicating a diverse sRNA set in *P. yezoensis*.

Although many sRNAs expressed in *P. yezoensis* remained unidentified, they were annotated and classified into noncoding RNAs (Rfam, GenBank), homologs of plant miRNAs (miRBase), or siRNAs according to their identity with sequences from these databases or their characteristics of being siRNA. In the case that some sRNAs were mapped to more than one category, the following priority rule was adopted: rRNA and so forth (in which GenBank > Rfam) > known miRNA > siRNA [27]. Only a few of the sRNAs were annotated; ~85% total reads and ~95% unique reads remained nonannotated. Of all the annotated sRNAs, siRNAs were the most abundant sequences retrieved from the *P. yezoensis* unique sRNA pools, with the highest read frequency of all sRNA categories in both samples: 4.15% for PYF and 1.84% for PYL (Table 1). Yet, in the total sRNA pools, siRNA represented a significant part (8.29%) in PYF, while rRNA (5.88%) and tRNA (5.59%) were the significant components in PYL. Homologs of known plant miRNAs accounted for ~0.4% and ~0.61% of the unique sequences in PYF and PYL, respectively; whereas in total sequence pools, the numbers were ~0.96% and ~2.69% in PYF and PYL, respectively, indicating that homologs of miRNA might be expressed differently in PYF and PYL. sRNAs mapped to snRNA and snoRNA were rare, and the remaining sRNAs were not annotated. Analysis of common and specific sequences showed that only ~6% of the unique sequences were shared by the two samples (Table 2), suggesting the presence of a diverse set of endogenous sRNAs in *P. yezoensis*. 

### 3.2. miRNAs in *P. yezoensis*

The identification of a diverse set of sRNAs in *P. yezoensis *prompted us to examine whether they were indeed functional. We used homologs of known plant miRNAs and the remaining nonannotated sRNAs to identify candidates of known and novel miRNA families in *P. yezoensis*, respectively (Figure 1). Finally, we identified 14 candidate miRNAs in *P. yezoensis*, including two mature miRNAs identified earlier [24].

**Figure 3:**
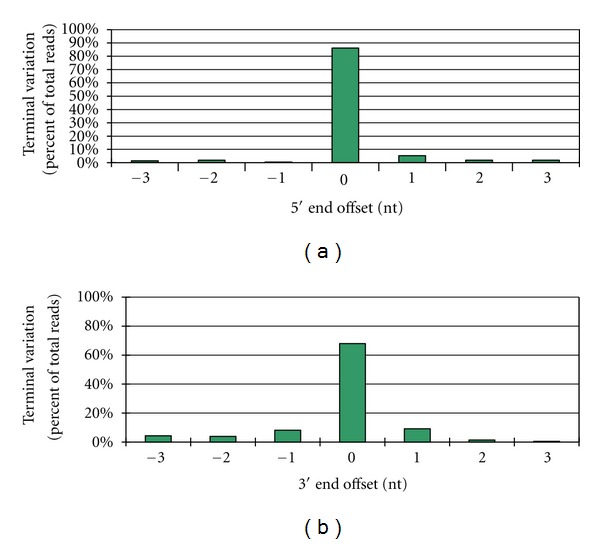
*P. yezoensis* miRNA sequence terminal variation analysis. Each unique sequence with 3′ terminal nucleotide deletion or 5′ terminal nucleotide extension corresponding to the mature miRNA selected is assigned a negative offset number, whereas the unique sequence with 3′ terminal nucleotide extension or 5′ terminal nucleotide deletion is assigned a positive offset number. In all cases, the percentage of heterogenicity for each unique *P. yezoensis* miRNA was obtained by dividing the read number of each variant by the total read number.

Each miRNA had a single precursor. The length of pre-miRNA ranged from 47 to 246 nt, with a mean of 135 nt (Table 3). The MFE range was from −135 to −20 kcal mol^−1^, with a mean of −65 kcal mol^−1^, similar to the computational prediction values of Arabidopsis miRNA precursors (−57 kcal mol^−1^) and much lower than that of tRNA (−27.3 kcal mol^−1^) and rRNA (−33 kcal mol^−1^) [32]. The 14 miRNAs were designated Pye-miR 1–14. Sequence terminal variation analysis showed that the percentage of length heterogeneity was ~14% for the 5′ end of *P. yezoensis* miRNAs and 32% for the 3′ end with either a 3 nt deletion or a 3 nt extension (Figure 3).

### 3.3. Expression Patterns of *P. yezoensis* miRNA Candidates during Different Generations

To investigate the likely roles of miRNAs in *P. yezoensis *development, we sequenced sRNAs from *P. yezoensis* sporophytes and gametophytes. A total of 14 miRNAs were identified, of which only 1 was sequenced in both samples, 7 were sequenced exclusively from PYF, and 6 were sequenced exclusively from PYL. This indicated that they might have an important role in *P. yezoensis *development. To determine the likely regulated genes, we predicted targets for these miRNAs on the basis of the rules for plant miRNA target prediction suggested by Allen et al. [30]. Most miRNAs found none or < 5 targets, except that 36 EST contigs were suggested as targets for Pye-miR4. However, the *P. yezoensis* genome is not sequenced and most ESTs are not annotated, so it is difficult to determine whether these miRNA targets have any functional bias.

### 3.4. siRNA in *P. yezoensis*

To identify siRNA in *P. yezoensis*, we compared tags from clean reads against each other. A pair of perfectly complementary sRNAs with a 2 nt overhang at the 3′-end was considered to be siRNA. Potential siRNAs were found to be expressed in *P. yezoensis* with 159,904 (4.15%) and 55,752 (1.84%) unique sequences; and 903,198 (8.29%) and 259,369 (2.16%) total sequences in PYF and PYL, respectively. Yet, owing to the lack of *P. yezoensis* genome information, we cannot determine their location. Thus, we cannot determine if they are phased relative to each other or if they have a role in silencing repetitive sequences in *P. yezoensis*, as for other organisms.

### 3.5. Homologs of Known Plant miRNAs in *P. yezoensis*

We compared all the clean reads with all known plant miRNAs available from miRBase (miRBase Sequence Database version 15; http://www.mirbase.org/) to identify homologs of known miRNAs. If a *P. yezoensis* sRNA exhibited homology with ≤2 mismatches (or 90% identity) with other known miRNAs, it was considered as a homolog of known miRNAs and these can be classified into 449 known miRNA families, allowing one or two mismatches between sequences (additional data file 1, see Supplementary Material available online at doi:10.1155/2012/912843). We compared them to miRNAs from 34 other species, including *Arabidopsis thaliana*,* Oryza sativa*,* Sorghum bicolor*,* Pinus taeda*,* Physcomitrella patens*, and *Chlamydomonas reinhardtii* (additional data file 1). Among these homologs, 56 were expressed in *A. thaliana*, 37 in *O. sativa*, 23 in *P. patens,* and only 4 in *C. reinhardtii*.

### 3.6. Experimental Validation of *P. yezoensis* miRNAs

**Figure 4:**
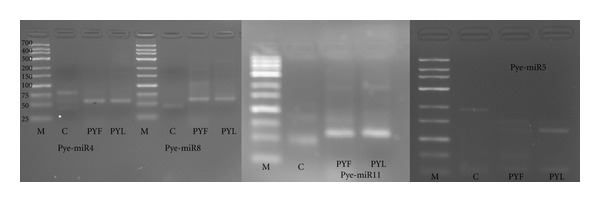
Experimental validation of some* P. yezoensis* miRNAs. M: marker; C: negative control.

We used RT PCR to detect the expression of *P. yezoensis* miRNAs and 4 were validated by PCR amplification. PCR products of the expected sizes (60–80 bp) were amplified (Figure 4), recovered, and sequenced, increasing confidence in their expression. Some larger PCR products might result from precursor RNAs. 

## 4. Discussion

sRNAs of 21 nt were the most abundant in PYF, consistent with an earlier report [24], whereas in PYL the length of enriched sRNAs is 22 nt. This is different from the case in *A. thaliana,* where 24 nt sRNAs represent a major part [33, 34]. Earlier reports indicated that *P. patens* and *C. reinhardtii* also lacked the enrichment of 24 nt sRNAs [35–37]. The length of sRNAs is determined by the species of enzymes that participate in their processing. For example, DCL2 produced sRNAs of 24 nt, whereas DCL1 produced sRNAs of 21 nt [37]. The lack of enrichment of 24 nt sRNAs in *P. yezoensis*, *C. reinhardtii,* and *P. patens* indicated that the RNA processing complexes in these lower photosynthetic organisms might differ from those of *A. thaliana. *The size of the most abundant sRNAs from PYF was different from that of PYL, indicating that RNA processing enzymes and level of expression might be different within species and even in different generations of the same organism.

To identify potential known miRNAs in *P. yezoensis*, we compared all sRNAs to all known plant miRNAs in miRBase and found quantity homologs; however, these homologs did not meet the criteria we used for miRNA precursor filtering. The most straightforward interpretation for this is the lack of genome information for *P. yezoensis*, although scenarios that *P. yezoensis* contains novel miRNAs that have no identifiable homologs in other organisms cannot be ruled out. The unicellular green algae *C. reinhardtii* lacks homologous miRNAs with other organisms and even with other green algae [37]. Thus, we proposed that *P. yezoensis* has novel miRNAs that have no sequence homology with others, as for *C. reinhardtii*. We used the remaining nonannotated sRNAs to identify potential novel miRNAs in *P. yezoensis*. In all, 14 miRNAs were identified, indicating that *P. yezoensis *does have novel miRNAs that lack sequence homology with other known miRNAs.

Two mature miRNAs, Pye-miR2 and Pye-miR13, were identified earlier and designated m0001 and m0005 [24], respectively. Notwithstanding, the length of the precursor of Pye-miR2 is different from that of m0001, designated by Liang et al. [24]. Interestingly, Pye-miR13, which was sequenced from sporophyte of *P. yezoensis* and designated m0005 by Liang et al. [24], was found in gametophyte but not sporophyte of *P. yezoensis* in this study. This indicated that *P. yezoensis *might contain more miRNAs, which cannot be identified effectively by high-throughput sequencing.

Some of the homologs of known miRNAs were highly expressed. For example, homologs of miR3445 were sequenced 21,384 times in PYF and homologs of miR1442, miR1154, and miR1211 were sequenced 144,756, 41,203, and 22,573 times in PYL, respectively. miR3445 was reported only in *Arabidopsis lyrata* and was not reported even in *A. thaliana*. In this study, we sequenced homologs of miR3445 nearly 20,000 times in PYF but only 9 times in PYL, indicating its important role in PYF. Interestingly, homologs of miR1442, which were sequenced only from salt-stressed but not drought-stressed or untreated libraries of *O. sativa* [38], were most abundant in PYL with only 1 read in PYF. As mentioned in Section 1, sporophytes enter and germinate in shells, whereas gametophytes live on static substrates of the intertidal zone and experience stress from various sources forms during low tide [7–9]. We proposed that homologs of miR1442 might have an important role in salt stress in PYL. Talmor-Neiman et al. [39] reported that miR1211 is expressed at a very low level in the *P. patens *gametophyte and they proposed that it might be expressed in other stages of development, such as in the sporophyte. However, we sequenced homologs of miR1211 nearly 20,000 times in PYL (gametophyte) but only 13 times in PYF (sporophyte), indicating that its role in *P. yezoensis* might be different from that in *P. patens*.

It was suggested that miRNA might participate in sexual differentiation in *Porphyra* [40]. In this study, we identified 14 miRNAs from *P. yezoensis* and only 1 was sequenced from both PYF and PYL; the others were sequenced exclusively from either PYF or PYL, indicating that different miRNAs are expressed and regulated gene expression between different generations of *P. yezoensis*. This might be a reason for the differences of EST between the two generations [10]. Besides, the two generations have the same genome but they differ in many aspects, including habitat. Sporophytes live in shells and experience little change of environment. By contrast, gametophytes live on static substrates of the intertidal zone and experience stress caused by strong light, high temperature, and desiccation during low tide [7–9], which might result in expression of stress response-related miRNAs, as mentioned above for homologs of miR1442. Consistent results have been reported for other species. Homologs of miR2119, which might be drought responsive [41], presented in gametophyte *Porphyra haitanensis* with 1,479,099 copies. In the study of *Phaeodactylum tricornutum*, we identified more miRNAs from nitrogen-limited and silicon-limited samples [42]. This difference of expression of miRNAs in two generations of *P. yezoensis *might result in their different tolerance of various stresses. In fact, the gametophyte is very tolerant of stress caused by strong light, high temperature, and desiccation, whereas the sporophyte is not.

We intended to identify the exact functions of *P. yezoensis* miRNAs. Different targets have been suggested but, owing to the lack of genome sequences, we cannot determine whether these miRNA targets have any functional bias. Additionally, we used criteria for plant miRNA target prediction to identify *P. yezoensis* miRNA targets; so some bona fide targets might have been missed because the interaction of miRNAs with mRNA in this lower photosynthetic organism might differ from that in higher plants. 

## 5. Conclusion

The results of this study show that *P. yezoensis *has a complex sRNA processing system containing novel miRNAs that have no identifiable homolog in other organisms and that might have important regulator roles in *P. yezoensis* development.

## Supplementary Material

Supplementary table: Homologs of Known Plant miRNAs in *P. yezoensis*. *P. yezoensis* miRNA homolgs share sequence homology with known miRNAs from other plants are indicated with ‘‘+”.

## Figures and Tables

**Table 1:** Categorization of *P*. *yezoensis* small RNAs.

Category	PYF				PYL			
	Unique sRNA	Percent (%)	Total sRNA	Percent (%)	Unique sRNA	Percent (%)	Total sRNA	Percent (%)
Total	3853350	100%	10896642	100%	3025076	100%	1.2*E* + 07	100%
miRNA	15440	0.40%	104081	0.96%	18342	0.61%	322689	2.69%
rRNA	30209	0.78%	197902	1.82%	43363	1.43%	705106	5.88%
siRNA	159904	4.15%	903198	8.29%	55752	1.84%	259369	2.16%
snRNA	133	0.00%	663	0.01%	230	0.01%	581	0.00%
snoRNA	176	0.00%	345	0.00%	282	0.01%	1797	0.01%
tRNA	18336	0.48%	215562	1.98%	21798	0.72%	669520	5.59%
Unannotated	3629152	94.18%	9474891	86.95%	2885309	95.38%	1*E* + 07	83.65%

**Table 2:** Common and specific small RNAs between PYF and PYL.

Class	Unique sRNA	Percent (%)	Total sRNA	Percent (%)
Total sRNAs	6501964	100.00%	22881336	100.00%
PYF and PYL	376462	5.79%	11112797	48.57%
PYF specific	3476888	53.47%	6738816	29.45%
PYL specific	2648614	40.74%	5029723	21.98%

**Table 3:** Characteristics of *P*. *yezoensis* pre-miRNA sequences.

Id	Mfe^a^	Length^b^	seq^c^
Pye-miR1	−104	227	ACGCGCACAGCCTCGCCAACCGCGACGTCCGCCATCGCCA
			CGACCGCCCCCGTCGACAGCTCAACGGTGGCGGCGGCGGG
			GAAGCACGCCGGGTTGTTGTCCGCGGCCGTGCCGTTGGCC
			CCCTCCGCGACATCGCCCGACTTGATCGCCGCGCCCGTGT
			ACACGCACAGCGGGTTGTCCACCTGTGAGATGGGGT*TGGA*
			*CTCGATGGTGTAGGTGA*GAAGGTGGCG

Pye-miR2	−34.9	76	GAAAACTCAG*GACAGCGACGACGACGACGACC*GCAAGCCG
			CGCCCGTCGCCGTCGTACTAGCTGGGCTGATGACAA

			CGTGTGCCGCCGTACATCCGAAGTCATCGAGCTGCAGTTCC
			AGGACATCTTCGTGCTGGCGGACGGCGCCTTCTCCATCCAC
Pye-miR3	−72	193	GTCAACCGTTACAAGAACACGGAGGGACGTGACGACCCTC
			GCCGCCTGGTGTACACCATTCCACGTGACCCCATGCTGG*TG*
			*CACGACCCGGTCCTGGCATT*GCTGCGCACC

Pye-miR4	−28.8	47	G*GCGACGGCGACGGCGGCGAC*GCGGCCGCCGACCGATGGC
			TGTCGTA

Pye-miR5	−74.4	138	TAGGCCTGCAGCGTCAGGGCGGGGTGTGTCGCGCCCCCCG
			CGGCACAAAAGCCGCCGTCCGCGGCTAGCGGGAGGGTGGC
			CGGCTTCGAAAGAGGCGTTGCTGAGC*GCGGGCGCGCAGAA*
			*GACTCGTT*CGGCGGCCGC

Pye-miR6	−71.2	134	TGGCAGCAGCGCCGAGGGCGATCGCCGCGCGGCCCACCGC
			CGACCCCCCCAGCTCCCCCCTCCGCCCGGCGACGATGGGC
			GTGGGCGTCAGCGGCGGCCGG*TGGAGGCTGGAGCGGTCAAC*
			*GCC*CGGGCACGCC

			GCCACCGCCG*TGGTGTAGGTGCTGGCACGGA*CGCCGCCAAC
			GACGATATCCCCCTGCAGCGTCTGGGGGTTGTACAACCCCG
			CCGAGGTGCCCGTGGAGACGGCGGTCACCACCGACGACGC
Pye-miR7	−135	246	GGCGTCGGCGGCCACGTCCAGCGCGTCGCCCACCCGCACC
			GACCGCAGCGGCGCCGCGCGGCCATTGATGTACACCAGGT
			GCCCCGGCGTGGCGGTGAGCGCGTGGCCGCTGCGGGTGGT
			GGCG

Pye-miR8	−117	226	GCGCGCCCCG*TCGGACGGGACGAGGGCAGCA*AGCCGGCGC
			TTATGGCCGCGCGCGGCCTCCTGCGTGTGCTGCGTCGGGG
			AGAGCGGCTCGAGGTCGCCGGCGAGCCTCGTCTGCATGGC
			CGCCAGCTCGTGCTTTGGGCATGCCCACTTGGGCACGATG
			CCGGAGCGCACCTTCACCTTCAGCGTGTCAGCCGCGTGCC
			CTTTCTCGTCGCAGGCGCCGCACGCT

Pye-miR9	−20.3	71	TGGTGGGTTGTTTCTCTGTGTGTTCTGGGTGCTACGCGCC
			*TAAGGTACGTAAAACCACTAC*ACCCCTTCCT

Pye-miR10	−62.2	153	GTGGCTGGTACACAACAAGTACACGCGCTCTGAGATGGG
			CCGGAAGGCAGTCCGTGCCGGCGTCAAGGCCATGTACGC
			GTACCTCGGCGTCACTGACCGCGAGCGCGATGACGACGT
			CGGCAG*TGGGACTGTCCTTGGCATCT*CTATTGGCCA

Pye-miR11	−79.4	141	ATCCTTGGCC*GCCTCGGTGAGGGCTCGGACC*TGGTCCAAG
			GCCTTAGCGAGCTTACTCTGGAGGTCAGAGATGACCTCCG
			CCTGGGTGGGGTCGTCCCCCCGCCCAGAGTCTGAGCCCAT
			CCCTTGCGTGGACGCAAGGAA
			

Pye-miR12	−38.8	79	AAAAGCCGCC*TGGGGATGGGCATTGAAGGC*GTCCGCGGCT
			TCCTGAAAGCGCTTTGTGCGCGTACCCCGCGCGGCCACA

			GCTGTCGCGTCACAGCTCCAGCGCGCGTGCGCGGGCCGCG
Pye-miR13	−51.2	83	ACGGCCGCGGCCTGTCATGGTCGCTTGGGCACTGATGCGG
			TGG

Pye-miR14	−24.2	72	CATTGCCAAC*CGCTTCGTGACTCTCGGCATG*GGAGAGAGCC
			GCGATTATGGATTGCGGAGAGAGGACAGTGG

^a^Minimum free energy (cal/mol) of pre-miRNAs, predicted by mfold.

^b^Length of pre-miRNAs.

^c^Sequence of pre-miRNAs, the mature miRNA was indicated in italics.

## Abbreviations

PYF: The sporophyte of *Porphyra yezoensis*
PYL: The gametophyte of *Porphyra yezoensis*.
